# Supplementary material for: Dissecting the role of the human microbiome in COVID-19 via metagenome-assembled genomes
Source: Nat Commun. 2022 Sep 6;13:5235. doi: 10.1038/s41467-022-32991-w (PMC9446638; doi:10.1038/s41467-022-32991-w)
Supplement: Supplementary file 1 — Supplementary Information [file 41467_2022_32991_MOESM1_ESM.pdf]

Dissecting the Role of the Human Microbiome in COVID-19 via Metagenome-assembled Genomes

Supplementary information

Shanlin Ke[1], Scott T. Weiss[1], Yang-Yu Liu[1]

[1]*Channing Division of Network Medicine, Department of Medicine, Brigham and Women's Hospital and Harvard Medical School, Boston, MA, 02115, USA.*

# Corresponding authors: Y.-Y.L. (yyl@channing.harvard.edu).

Supplementary Figures and Tables

Fig. S1 The completeness and contamination of the 11,584 recovered MAGs.

Fig. S2 Basic characteristics of the 11,584 recovered MAGs and 5,403 nrMAGs from 514 microbiome samples in the discovery cohorts.

Fig. S3 The percentage of unknown SGBs identified from the recovered genomes.

Fig. S4 The relative contribution from each sample to the recovered MAGs and nrMAGs.

Fig. S5 The phylum information of nrMAGs.

Fig. S6 Alpha diversity of COVID-19 related human microbiome sample in the discovery cohort.

Fig. S7 Alpha diversity analyses of COVID-19 related human microbiome samples in the three validation cohorts.

Fig. S8 Beta diversity of COVID-19 related human microbiome samples in four discovery cohorts.

Fig. S9 Beta diversity analyses of COVID-19 related human microbiome samples in the three validation cohorts.

Fig. S10 COVID-19 related changes in strain richness of microbial species in the three validation cohorts.

Fig. S11 Venn diagram of top-30 species with the highest strain richness identified from different cohorts.

Fig.S12. Venn diagram of top-30 species with the highest COVID-19 related strain-richness change identified from different cohorts.

Fig. S13 The nrMAG-based classification model distinguishes COVID-19 from Non-COVID-19 controls.

Fig. S14 Cross-validation of the machine learning model between the two discovery cohorts.

Fig. S15 Heat map of top-30 most important nrMAGs related to the performance of cross-validation.

Fig. S16 External validation of the machine learning model.

Fig. S17 Sample collection timeline in the study of Yeoh et al.

Fig. S18 The permissive and protective nrMAGs of SARS-COV-2 infection identified on the study of Yeoh et al. showed similar distribution on the study of Zuo et al.
Fig. S19 KEGG module completeness of permissive and protective nrMAGs.
Fig. S20. The phylogenetic tree of strains (nrMAGs) that have the potential to use the pentose phosphate pathway (Pentose phosphate cycle, M00004, module completeness: 87.5%).
Fig. S21. The phylogenetic tree of strains (nrMAGs) that have the potential to use the pentose phosphate pathway (oxidative phase, glucose 6P => ribulose 5P, M00006, module completeness: 100%).
Fig. S22 Abundance comparison of the pentose phosphate pathway between COVID-19 patients and Non-COVID-19 controls.

Table S1 Validation cohorts analyzed in this study.

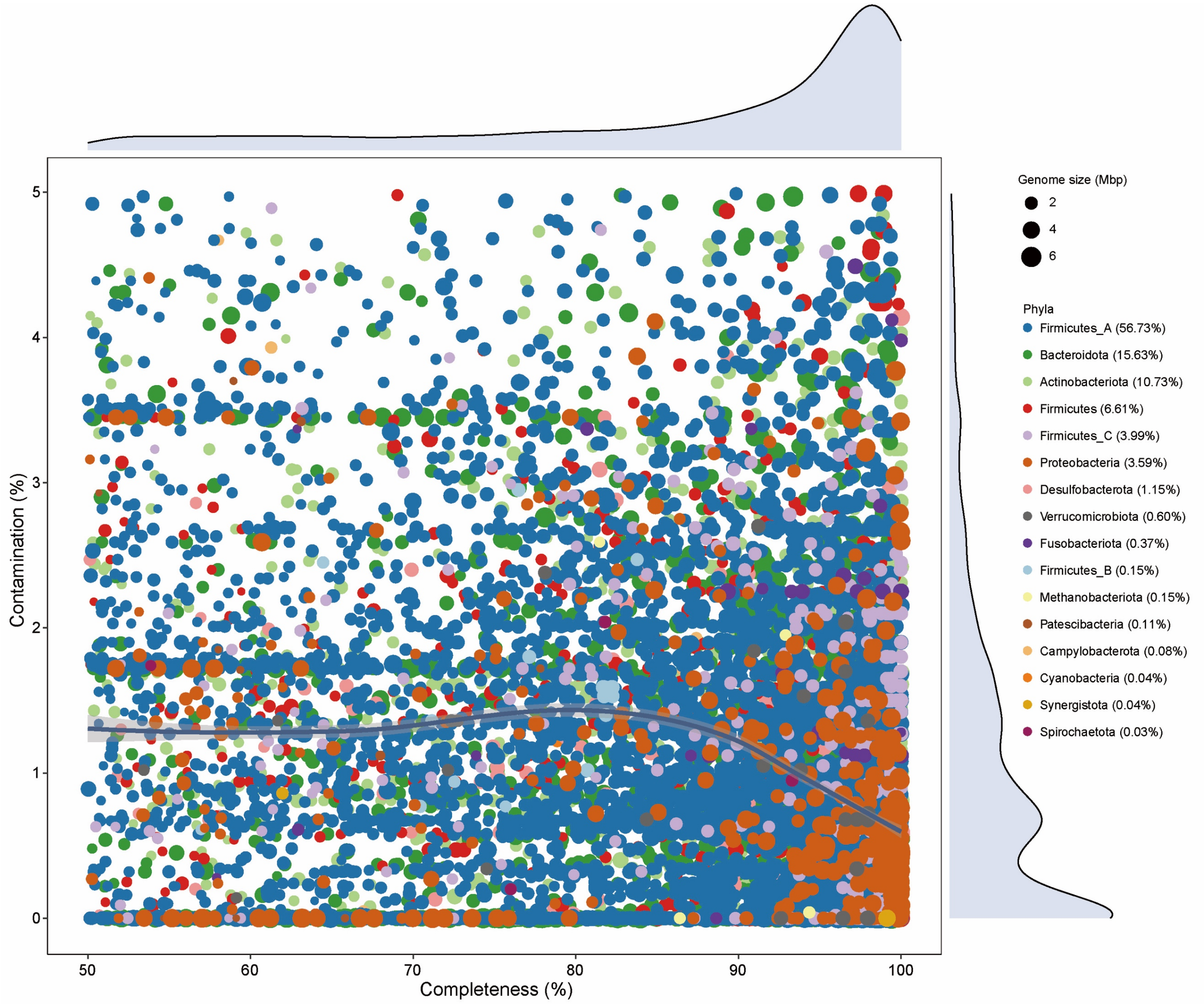

Fig. S1 The completeness and contamination of the 11,584 recovered MAGs. The color of each dot (MAG) represents its phylum and the size represents its genome size.

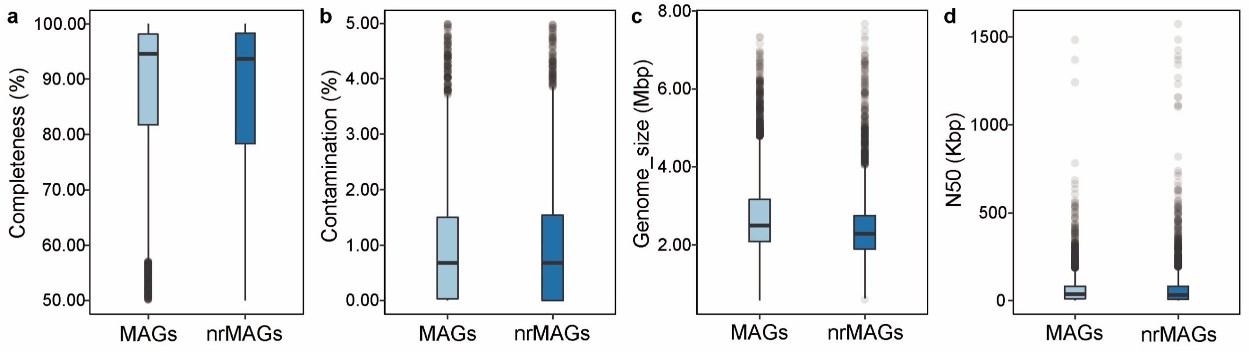

Fig. S2 Basic characteristics of the 11,584 recovered MAGs and 5,403 nrMAGs from 514 microbiome samples in the discovery cohorts. Boxplot of basic characteristics of MAGs and nrMAGs on completeness (**a**), contamination (**b**), genome size(**c**), and N50 (**d**). Boxplots with medians are shown; the lower and upper hinges correspond to the first and third quartiles (the 25th and 75th percentiles); the upper and lower whiskers extend from the hinge to the largest and smallest value no further than 1.5× interquartile range from the hinge; outliers are plotted by translucent circles.

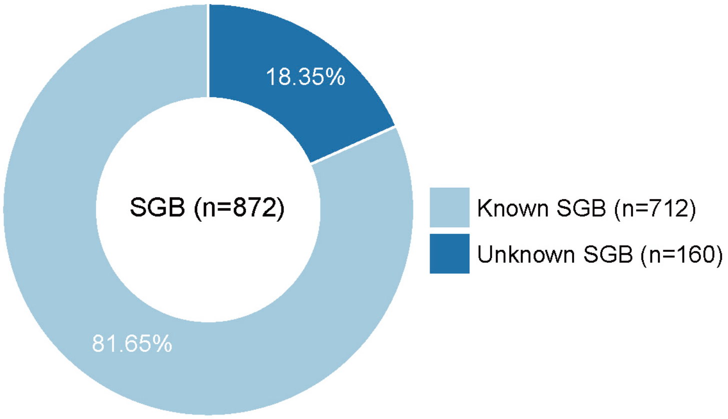

Fig. S3 The percentage of unknown SGBs identified from the recovered genomes. SGBs containing at least one reference genome (or metagenome-assembled genome) in the Genome Taxonomy Database (GTDB) were considered as known SGBs.

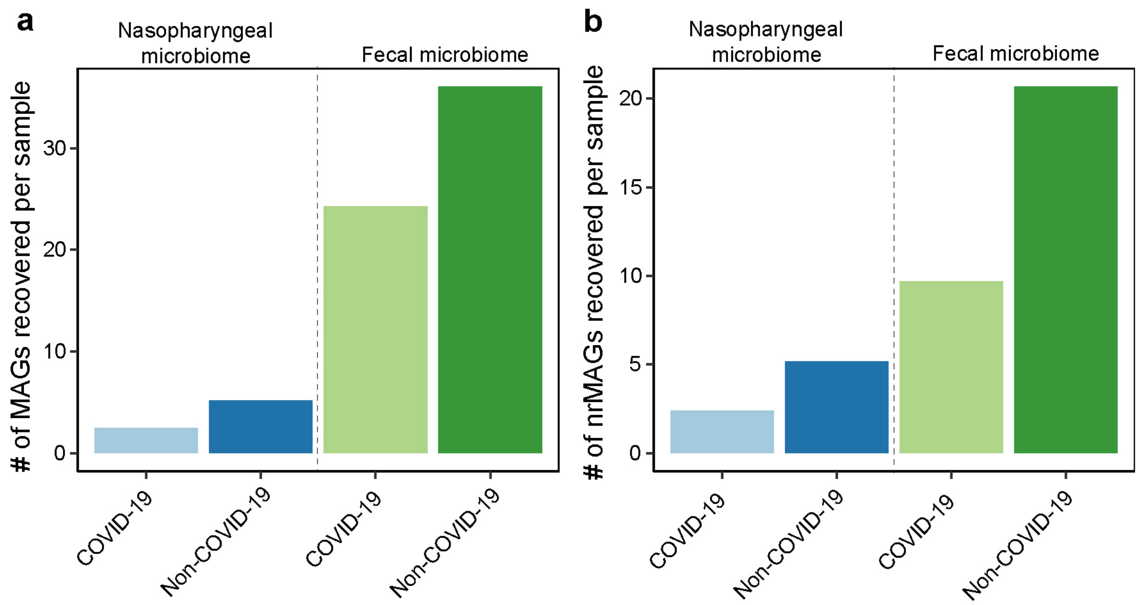

Fig. S4 The relative contribution from each sample to the recovered MAGs and nrMAGs. The normalized number of MAGs (**a**) and nrMAGs (**b**) recovered from different sample types and disease status.

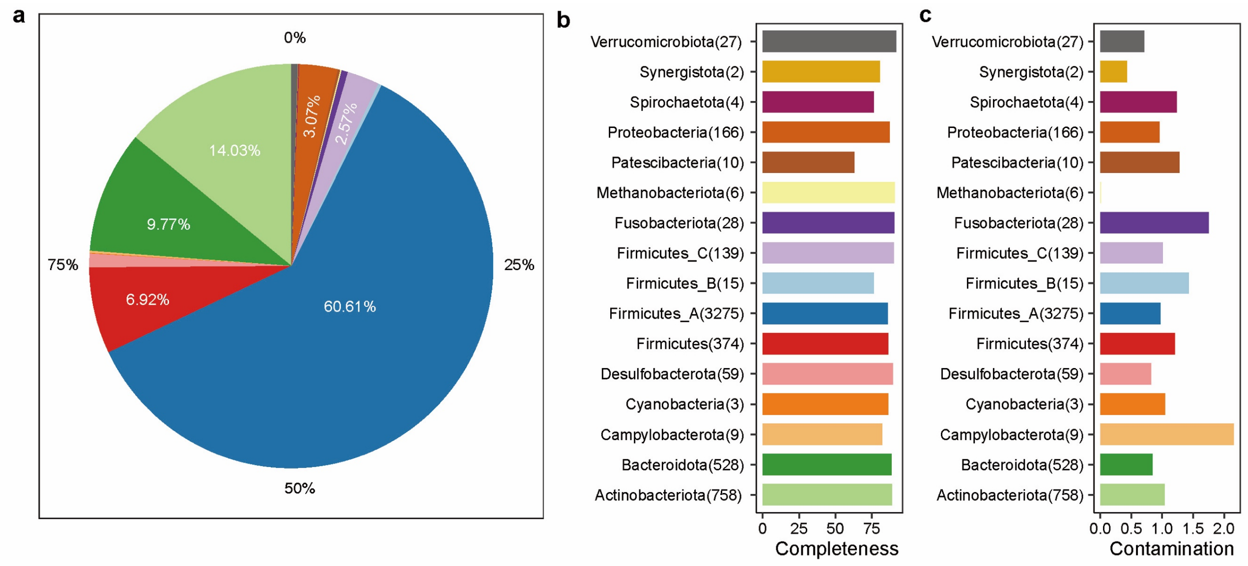

Fig. S5 The phylum information of nrMAGs. a, Pie chart shows the percentage of nrMAGs at the phylum level. The completeness (**b**) and contamination (**c**) of nrMAGs in different phyla.

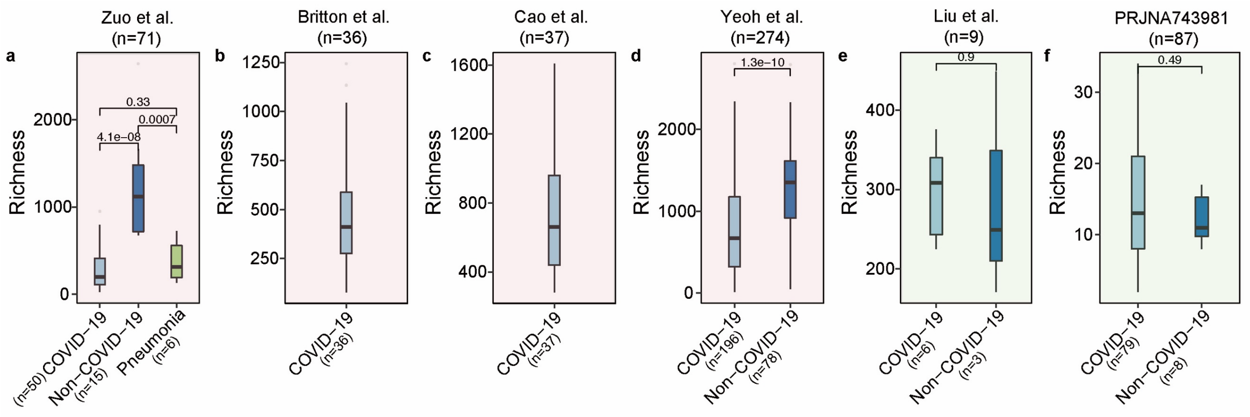

Fig. S6 Alpha diversity of COVID-19 related human microbiome sample in the discovery cohort. Richness (number of nrMAGs) of human microbiome at the nrMAG-level from the study of Zuo et al. (**a**), Britton et al. (**b**), Cao et al. (**c**), Yeoh et al. (**d**), Liu et al. (**e**), and PRJNA743981 (**f**). *P* values were calculated by two-sided Wilcoxon–Mann–Whitney test. Boxplots with medians are shown; the lower and upper hinges correspond to the first and third quartiles (the 25th and 75th percentiles); the upper and lower whiskers extend from the hinge to the largest and smallest value no further than 1.5× interquartile range from the hinge; outliers are plotted by translucent circles.

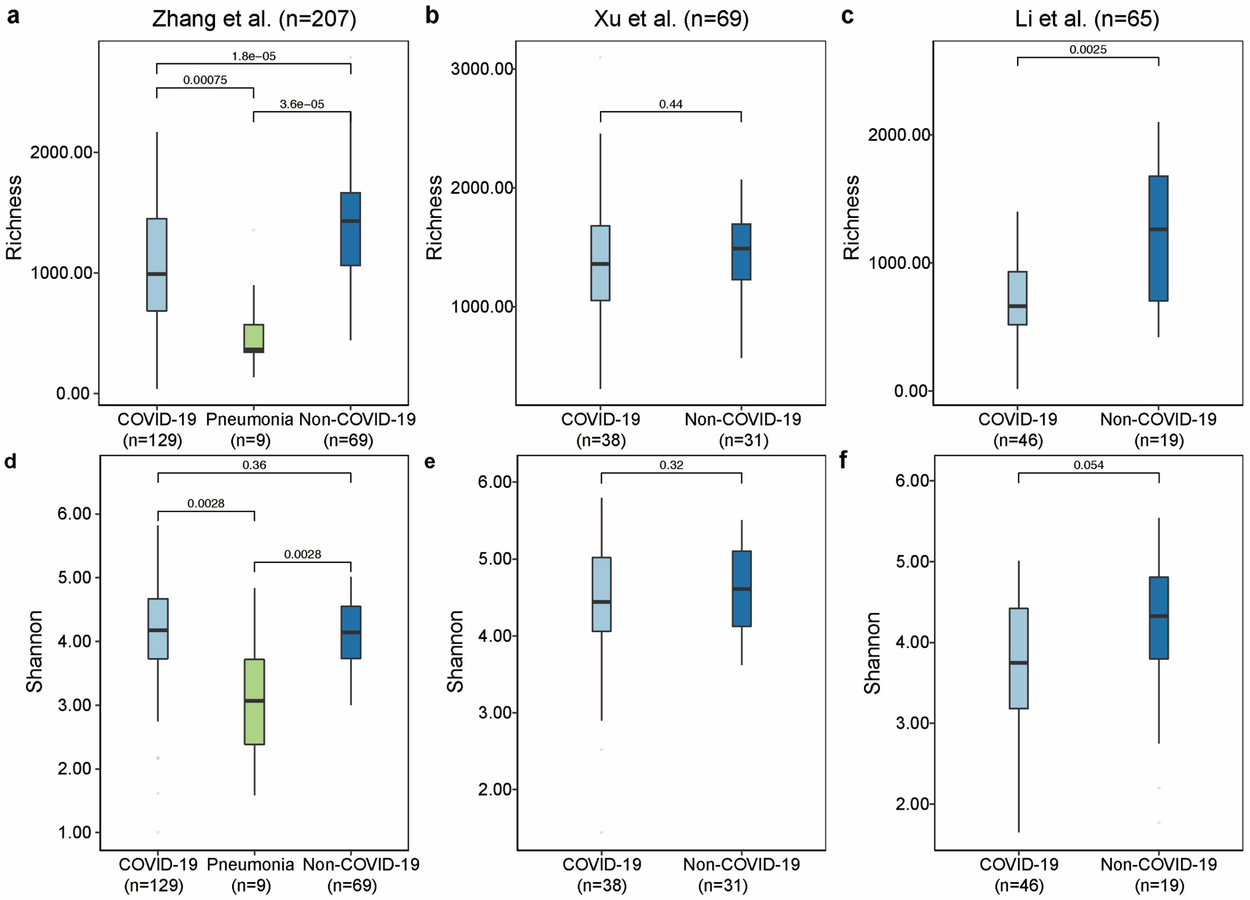

Fig. S7 Alpha diversity analyses of COVID-19 related human microbiome samples in the three validation cohorts. a-c: Richness of the human microbiome at the nrMAG-level from the study of Zhang et al. (a), Xu et al. (b), and Li et al. (c). **d-f**: Shannon index of the human microbiome at the nrMAG-level from the study of Zhang et al. (d), Xu et al. (e), and Li et al. (f). *P* values were calculated by two-sided Wilcoxon–Mann–Whitney test. Boxplots with medians are shown; the lower and upper hinges correspond to the first and third quartiles (the 25th and 75th percentiles); the upper and lower whiskers extend from the hinge to the largest and smallest value no further than 1.5× interquartile range from the hinge; outliers are plotted by translucent circles.

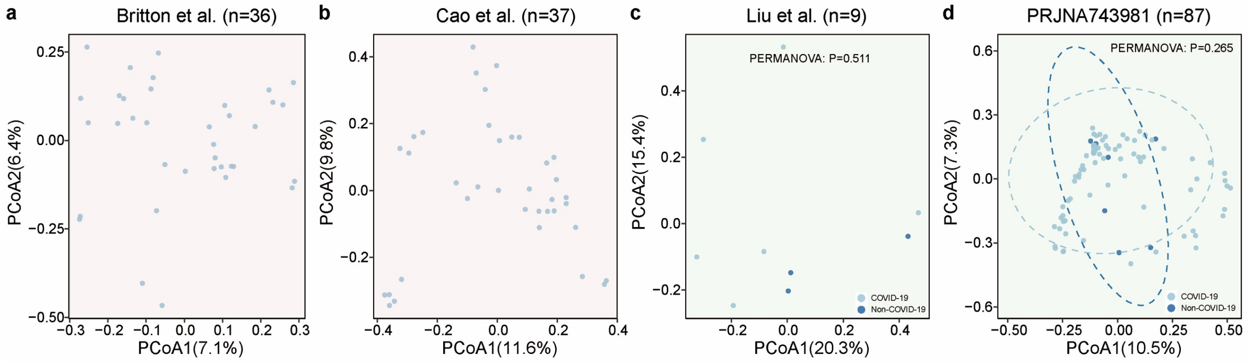

Fig. S8 Beta diversity of COVID-19 related human microbiome samples in four discovery cohorts. Principal Coordinates Analysis (PCoA) plot based on Bray–Curtis dissimilarity of microbial compositions from the study of Britton et al. (**a**), Cao et al. (**b**), Liu et al. (**c**), and PRJNA743981 (**d**). All PERMANOVA tests were performed with 9999 permutations based on Bray–Curtis dissimilarity, two-sided. The background color of each panel represents the source of the microbiome samples from the human gut (light red, **a**-**b**) or nasopharynx (light green, **c**-**d**).

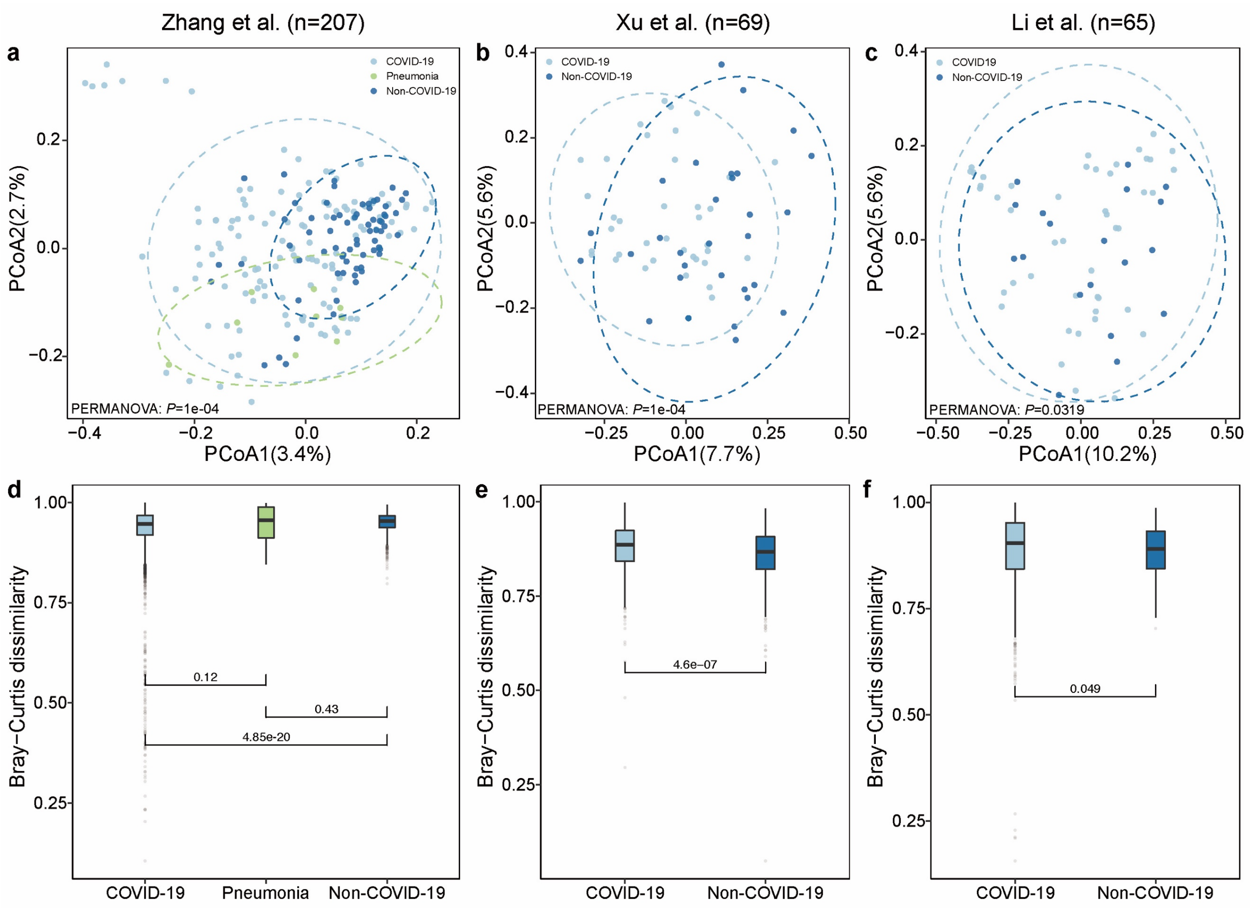

Fig. S9 Beta diversity analyses of COVID-19 related human microbiome samples in the three validation cohorts. a-c: Principal Coordinates Analysis (PCoA) plot based on Bray–Curtis dissimilarity of microbial compositions from the study of Zhang et al. (a), Xu et al. (b), and Li et al. (c). All PERMANOVA tests were performed with 9999 permutations based on Bray–Curtis dissimilarity, two-sided. **d-f**: Within-group Bray-Curtis dissimilarity of the human microbiome at the nrMAG-level from the study of Zhang et al. (d), Xu et al. (e), and Li et al. (f). *P* values were calculated by two-sided Wilcoxon–Mann–Whitney test. Boxplots with medians are shown; the lower and upper hinges correspond to the first and third quartiles (the 25th and 75th percentiles); the upper and lower whiskers extend from the hinge to the largest and smallest value no further than 1.5× interquartile range from the hinge; outliers are plotted by translucent circles.

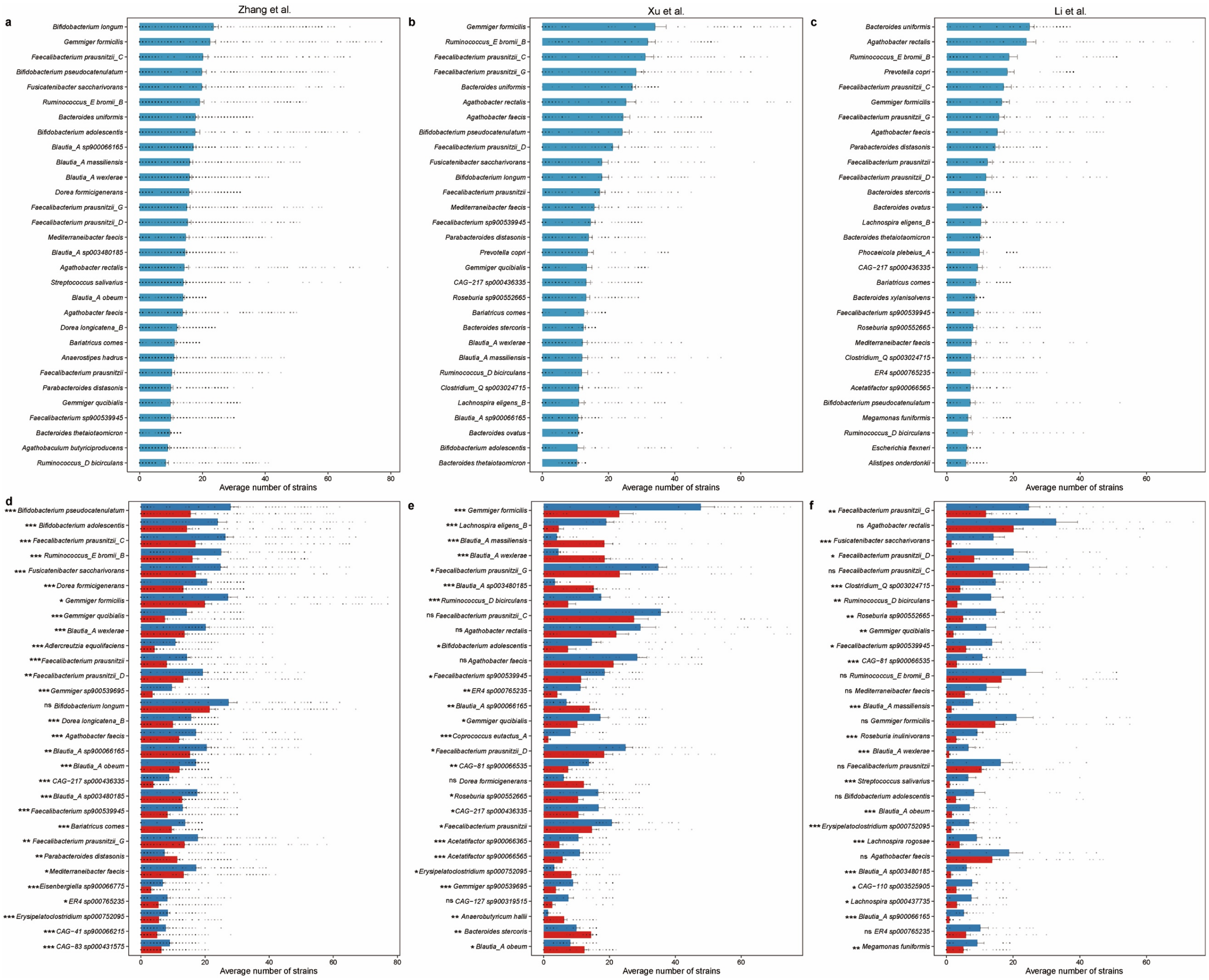

Fig. S10 COVID-19 related changes in strain richness of microbial species in the three validation cohorts. a-c: The top-30 species with the highest strain richness (i.e., the number of nrMAGs) identified from the study of Zhang et al. (a, sample size n = 198), Xu et al. (b, sample size n = 69), and Li et al. (c, sample size n = 65). **d-f**: The top-30 species with the highest strain richness change between the Non-COVID-19 (blue) and COVID-19 (red) samples identified from the study of Zhang et al. (d), Xu et al. (e), and Li et al. (f). Data are presented as mean ± standard error of mean. *P* values were calculated by two-sided Wilcoxon–Mann–Whitney test (ns: nonsignificant; *: $P<0.05$; **: $P<0.01$, ***: $P<0.001$).

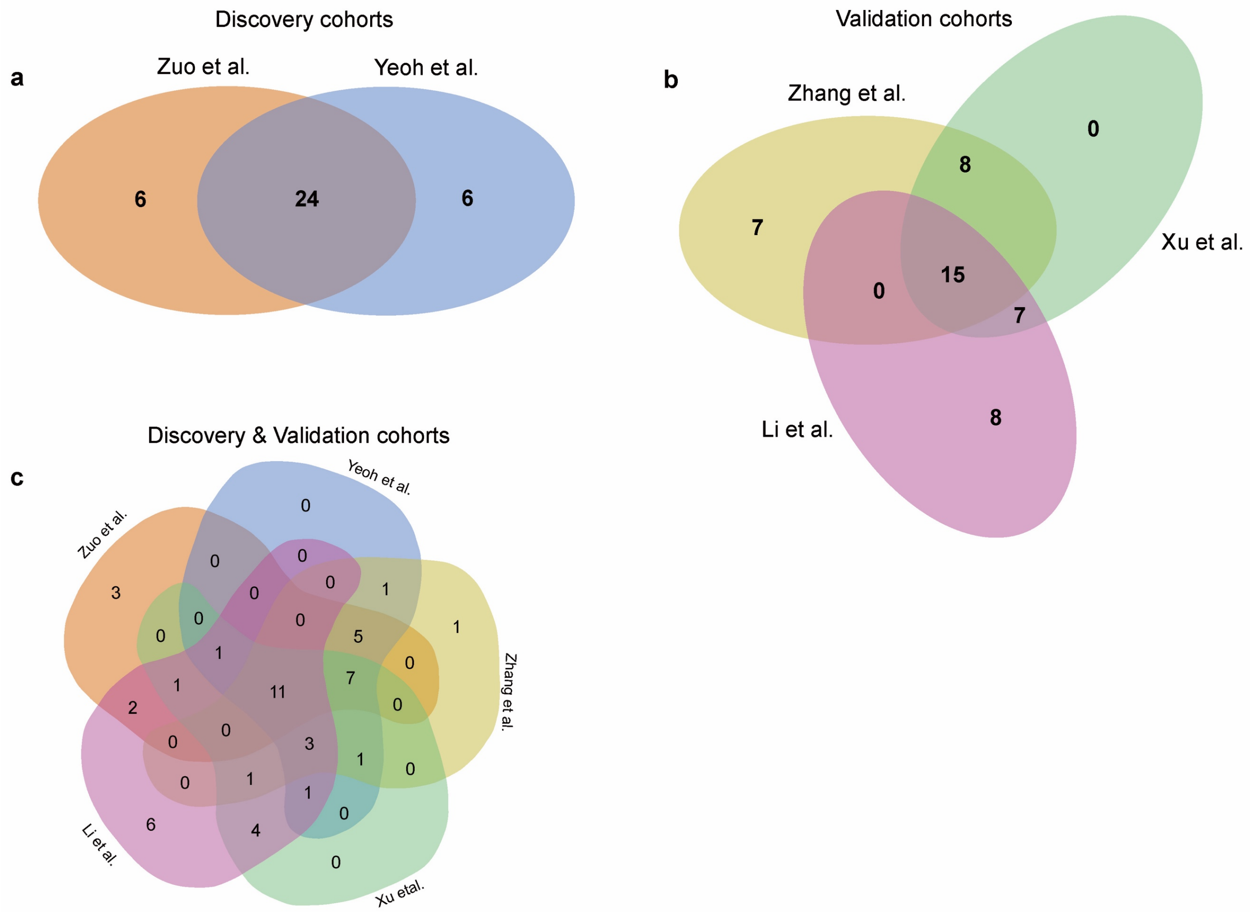

Fig. S11 Venn diagram of top-30 species with the highest strain richness identified from different cohorts. a, two discovery cohorts. **b**, three validation cohorts. **c**, two discovery and three validation cohorts.

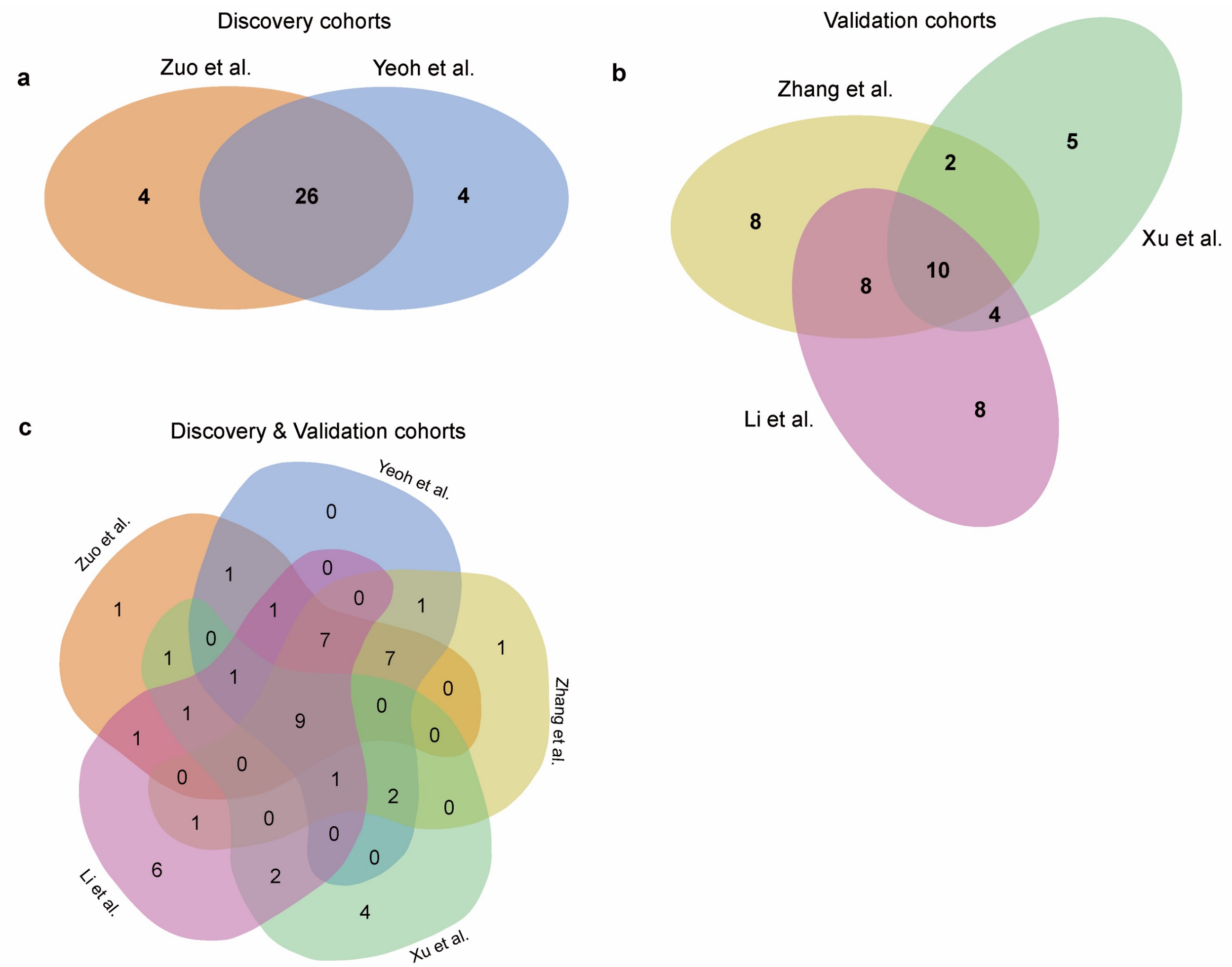

Fig.S12. Venn diagram of top-30 species with the highest COVID-19 related strain-richness change identified from different cohorts. a, two discovery cohorts. **b**, three validation cohorts. **c**, two discovery and three validation cohorts.

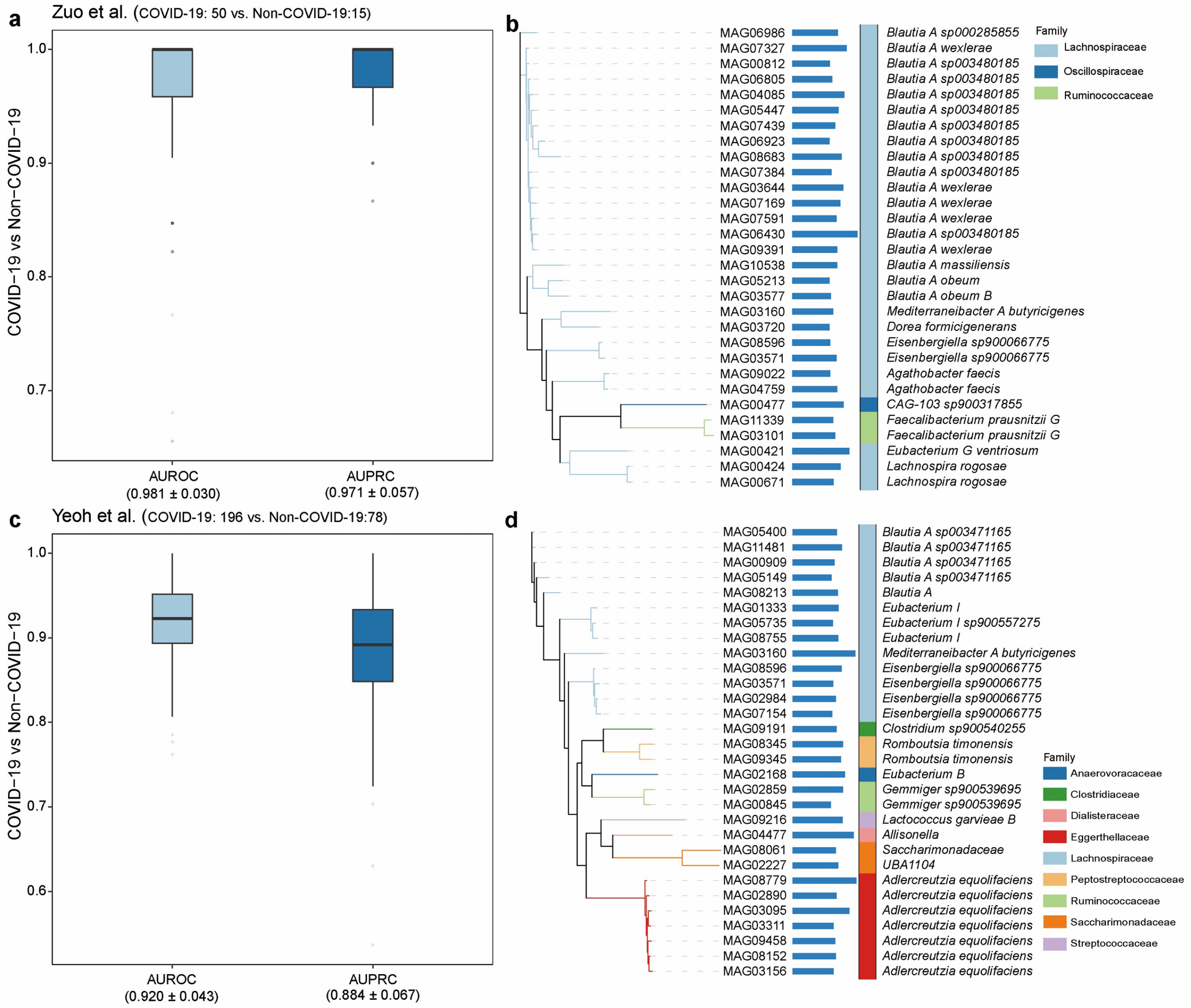

Fig. S13 The nrMAG-based classification model distinguishes COVID-19 from Non-COVID-19 controls. The classification performance of patients with COVID-19 vs Non-COVID-19 healthy controls in the study of Zuo et al. (**a**, sample size n = 65) and Yeoh et al. (**c**, sample size n = 274). Data are presented as mean ± standard deviation. The top 30 important nrMAGs related to performance of classification identified from Zuo et al. (**b**) and Yeoh et al (**d**). The importance of each feature was quantified by the Mean Decrease in Accuracy (MDA) of the classifier due to the exclusion (or permutation) of this feature. The length of horizontal bar represents the mean MDA value. The colors of vertical bar represent the taxonomy information of nrMAGs at the family level. The phylogenetic tree of these nrMAGs was constructed using PhyloPhlAn (https://huttenhower.sph.harvard.edu/phylophlan/). Boxplots with medians are shown; the lower and upper hinges correspond to the first and third quartiles (the 25th and 75th percentiles); the upper and lower whiskers extend from the hinge to the largest and smallest value no further than 1.5× interquartile range from the hinge; outliers are plotted by translucent circles.

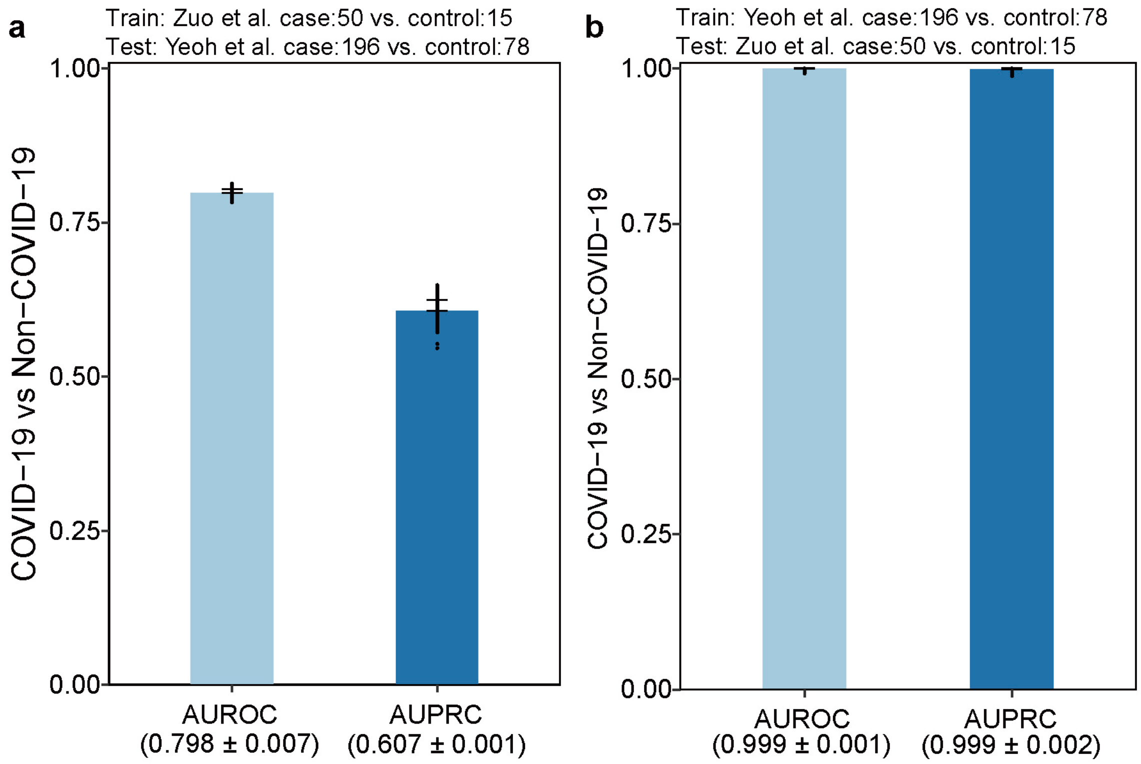

Fig. S14 Cross-validation of the machine learning model between the two discovery cohorts. **a**, The machine learning model was trained on the data from Zuo et al. (sample size n = 65) and then tested on the data from Yeoh et al. (sample size n = 274). **b**, The machine learning model was trained on the data from Yeoh et al. (sample size n = 274) and then tested on the data from Zuo et al. (sample size n = 65). The bars represent mean ± standard deviation of two standard classification performance metrics: the Area Under the Receiver Operating Characteristic (AUROC) curve, and the Area Under the Precision-Recall Curve (AUPRC).

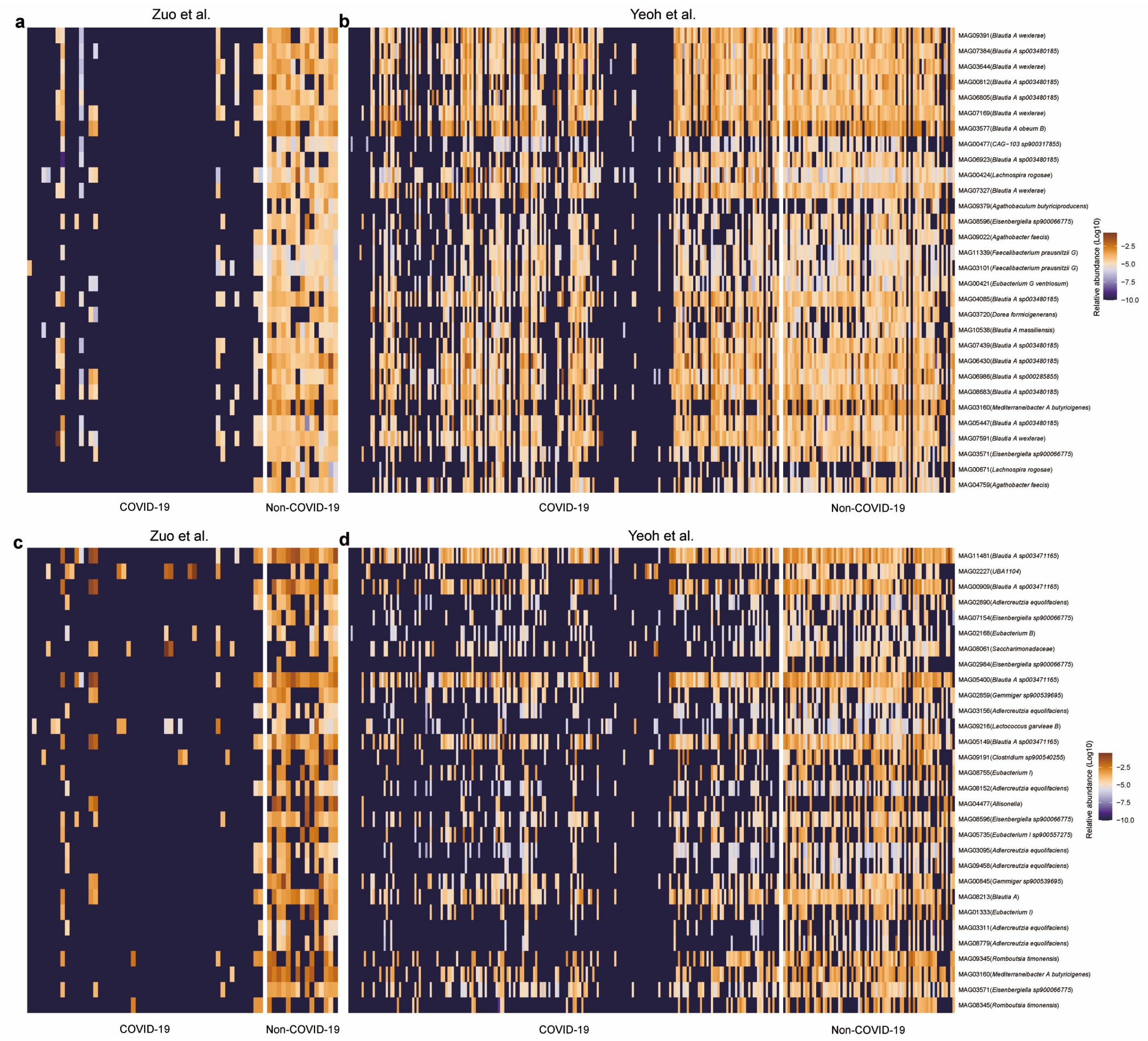

Fig. S15 Heat map of top-30 most important nrMAGs related to the performance of cross-validation. a,b: The relative abundances of the most important features in the machine learning model (trained on the data from Zuo et al.) in the microbiome samples of Zuo et al. (a) and Yeoh et al. (b). **c,d**: The relative abundances of the most important features in the machine learning model (trained on the data from Yeoh et al.) in the microbiome samples of Zuo et al. (c) and Yeoh et al. (d). The importance of each feature was quantified by the Mean Decrease in Accuracy (MDA) of the classifier due to the exclusion (or permutation) of this feature.

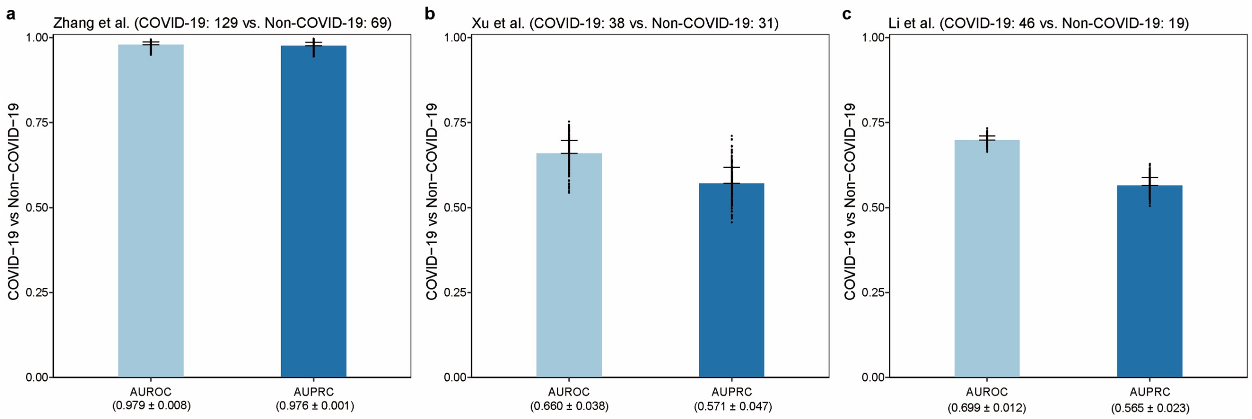

Fig. S16 External validation of the machine learning model. The machine learning model was trained on the data from Yeoh et al. and then tested on the data from Zhang et al. (**a**, sample size n = 198), Xu et al. (**b**, sample size n = 69), and Li et al (**c**, sample size n = 65). The bars represent mean ± standard deviation of two standard classification performance metrics: the Area Under the Receiver Operating Characteristic (AUROC) curve, and the Area Under the Precision-Recall Curve (AUPRC).

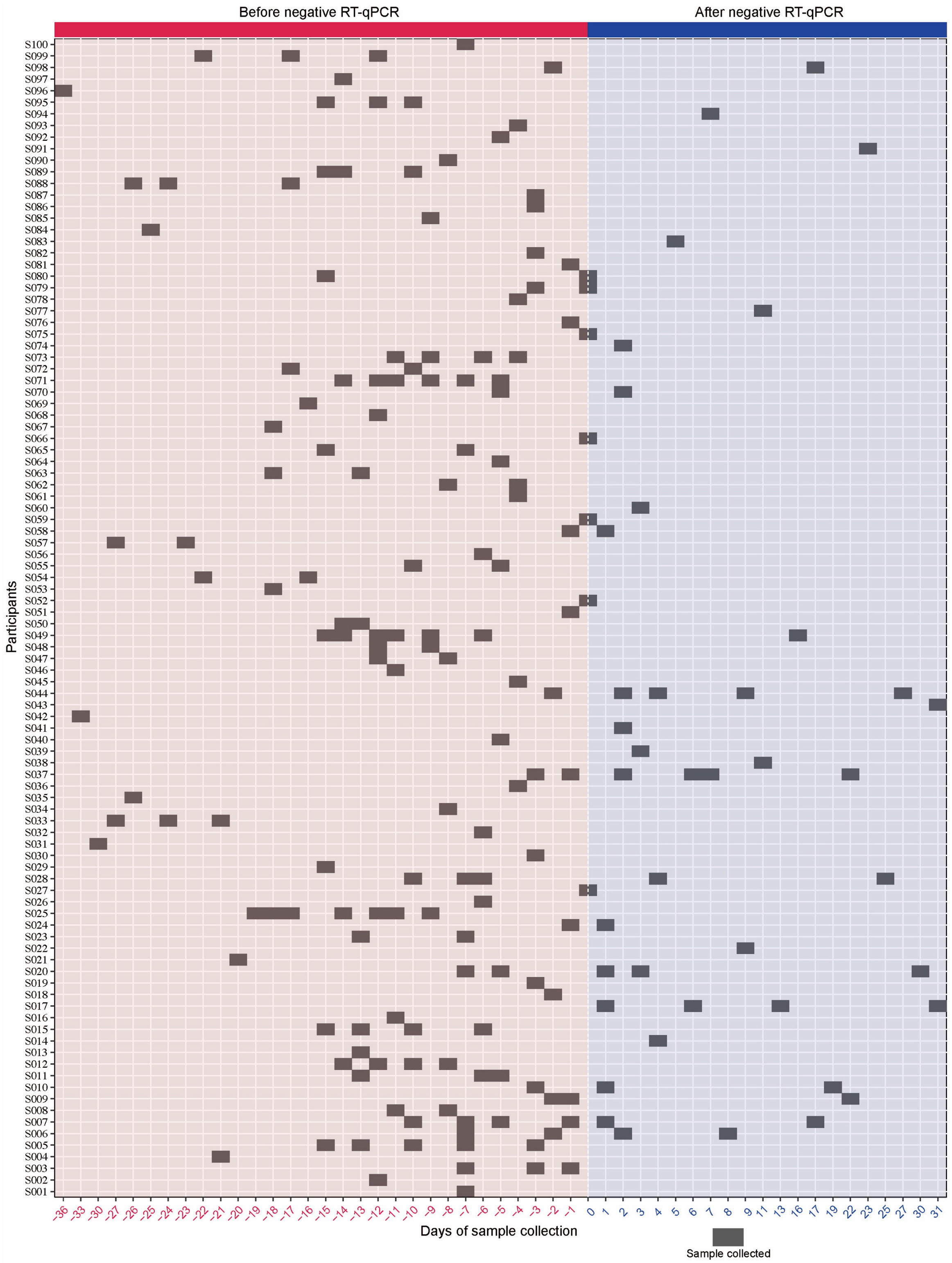

Fig. S17 Sample collection timeline in the study of Yeoh et al. Relative timeline (days) of microbiome samples collected before or after (⩾ 0 days) negative RT-qPCR result from COVID-19 patients.

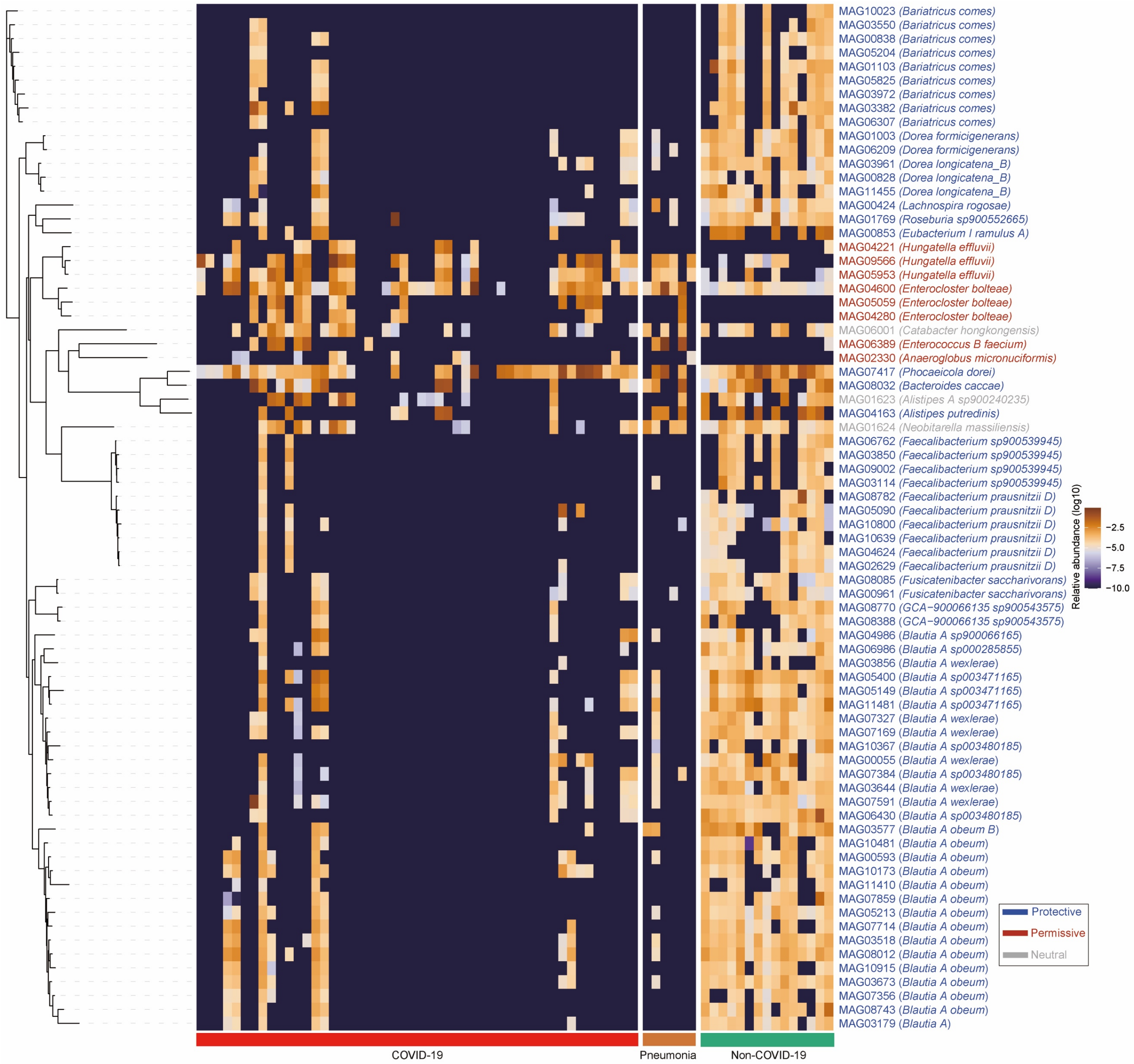

Fig. S18 The permissive and protective nrMAGs of SARS-COV-2 infection identified on the study of Yeoh et al. showed similar distribution on the study of Zuo et al. The heat map showed the abundance distribution of permissive, neutral, and protective nrMAGs on the study of Zuo et al. These nrMAGs were taxonomically annotated using GTDB-Tk based on the Genome Taxonomy Database. The colors of the taxonomical label represent permissive, protective, or neutral nrMAGs. The phylogenetic tree of these nrMAGs was constructed using PhyloPhlAn (https://huttenhower.sph.harvard.edu/phylophlan/).

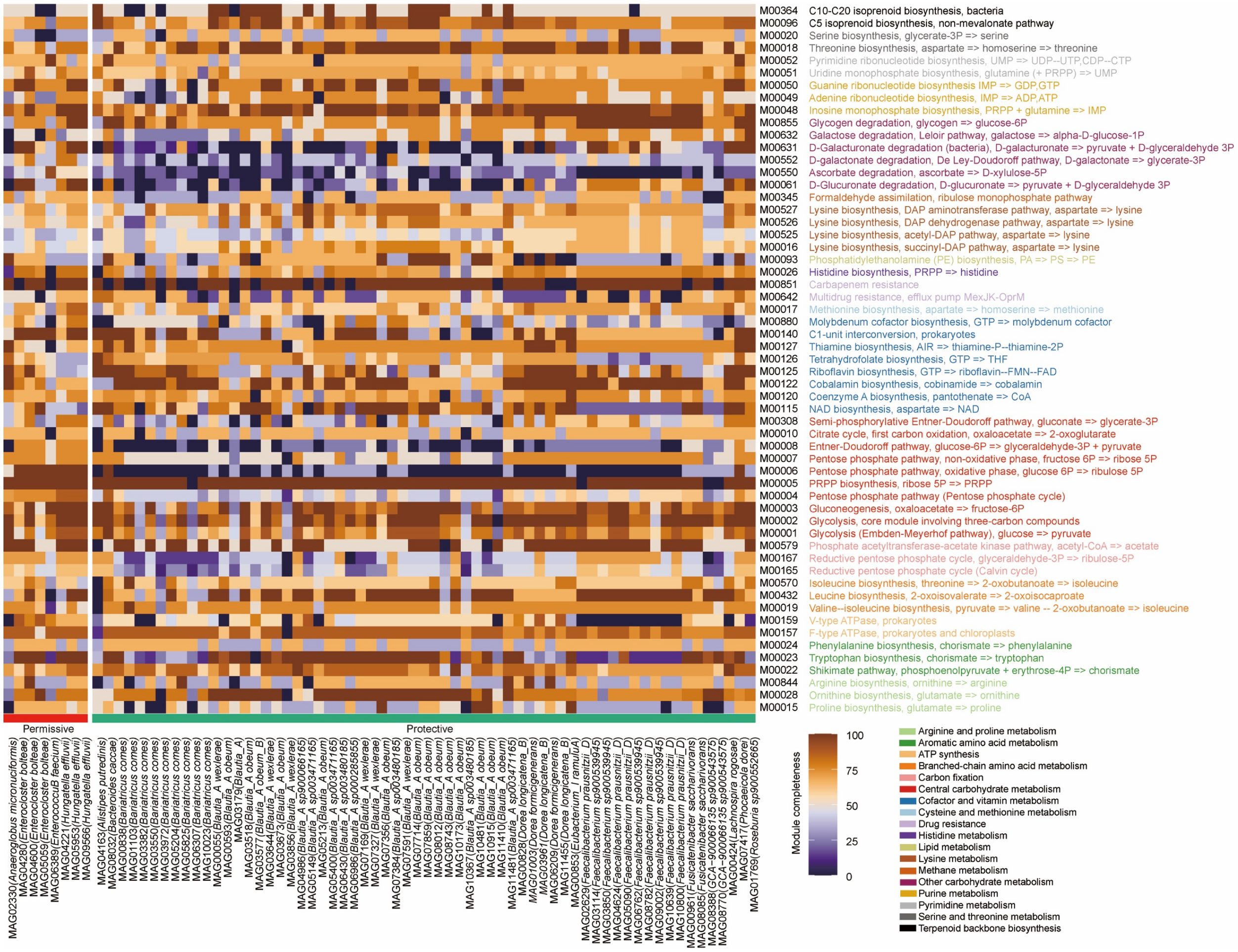

Fig. S19 KEGG module completeness of permissive and protective nrMAGs. The heat map showed the KEGG module with at least 50% completeness. The different colors of the module's name indicate which pathway they belong to (lower legend).

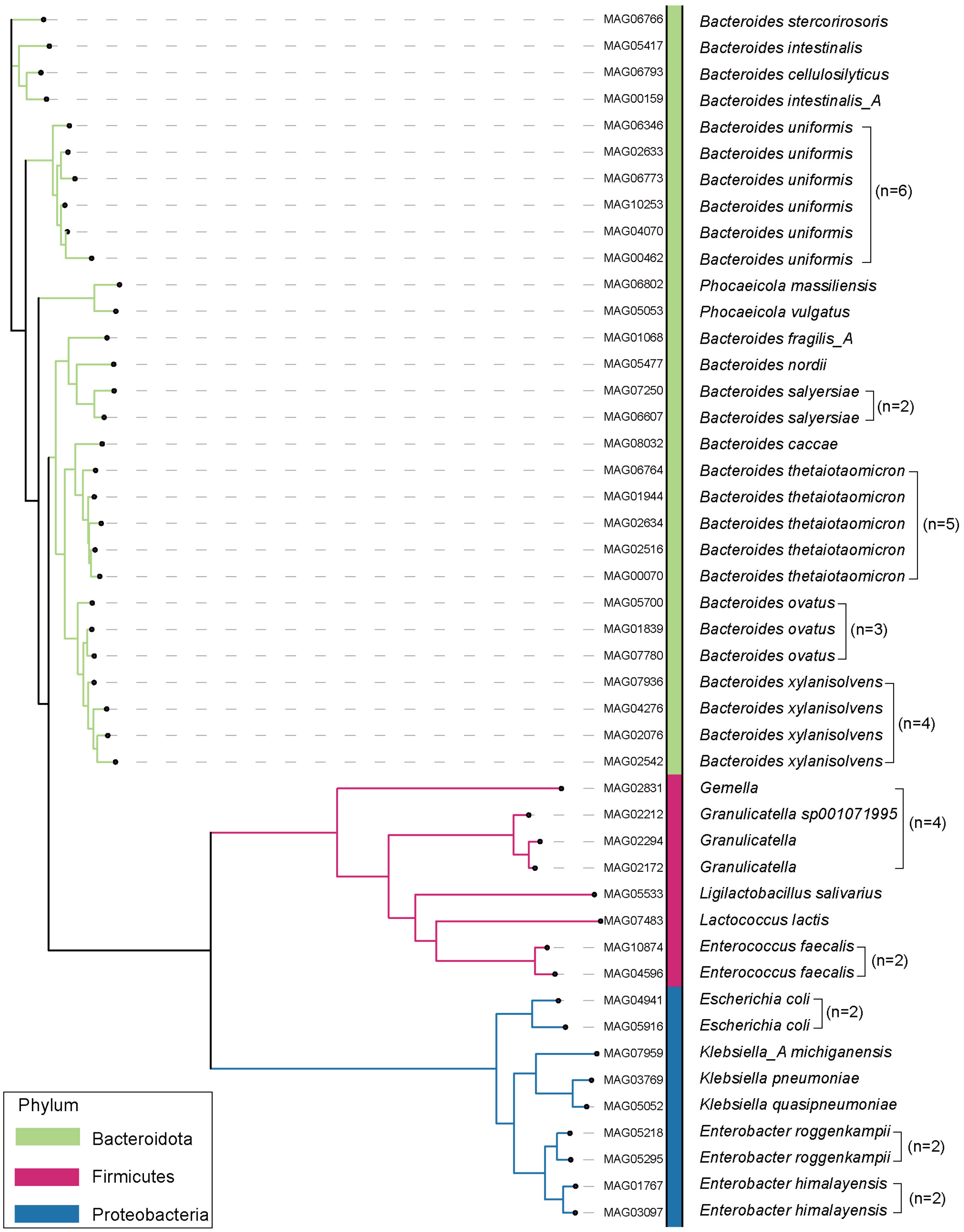

Fig. S20. The phylogenetic tree of strains (nrMAGs) that have the potential to use the pentose phosphate pathway (Pentose phosphate cycle, M00004, module completeness: 87.5%). The phylogenetic tree of nrMAGs was constructed using PhyloPhlAn (https://huttenhower.sph.harvard.edu/phylophlan/) and visualized using iTOL (https://itol.embl.de/). The color of cycle and clades represents phylum.

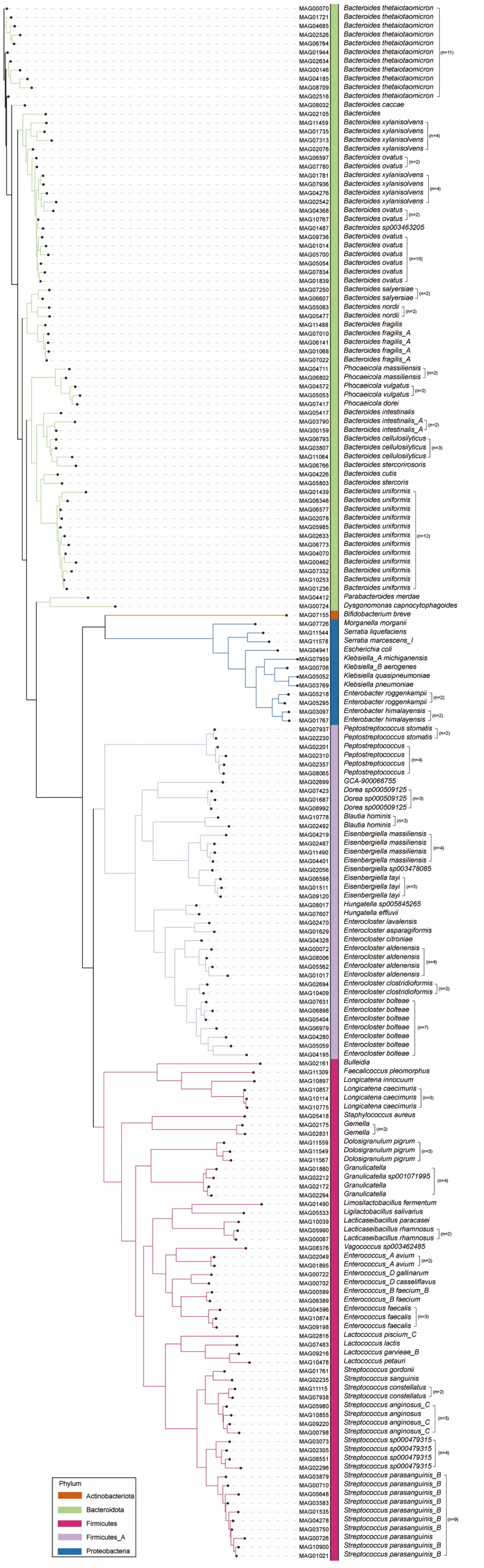
MAG00070 Bacteroides thetaiotaomicron
MAG01721 Bacteroides thetaiotaomicron
MAG04685 Bacteroides thetaiotaomicron
MAG02526 Bacteroides thetaiotaomicron
MAG06764 Bacteroides thetaiotaomicron
MAG01944 Bacteroides thetaiotaomicron
MAG02634 Bacteroides thetaiotaomicron
MAG00146 Bacteroides thetaiotaomicron
MAG04185 Bacteroides thetaiotaomicron
MAG08709 Bacteroides thetaiotaomicron
MAG02516 Bacteroides thetaiotaomicron
(n=11)
MAG08032 Bacteroides caccae
MAG02105 Bacteroides
MAG11459 Bacteroides xylanisolvens
MAG01735 Bacteroides xylanisolvens
MAG07313 Bacteroides xylanisolvens
MAG02076 Bacteroides xylanisolvens
(n=4)
MAG06597 Bacteroides ovatus
MAG07780 Bacteroides ovatus
(n=2)
MAG01781 Bacteroides xylanisolvens
MAG07936 Bacteroides xylanisolvens
MAG04276 Bacteroides xylanisolvens
MAG02542 Bacteroides xylanisolvens
(n=4)
MAG04368 Bacteroides ovatus
MAG10767 Bacteroides ovatus
(n=2)
MAG01487 Bacteroides sp003463205
MAG09736 Bacteroides ovatus
MAG01014 Bacteroides ovatus
MAG05700 Bacteroides ovatus
MAG05054 Bacteroides ovatus
MAG07834 Bacteroides ovatus
MAG01839 Bacteroides ovatus
(n=10)
MAG07250 Bacteroides salyersiae
MAG06607 Bacteroides salyersiae
(n=2)
MAG05083 Bacteroides nordii
MAG05477 Bacteroides nordii
(n=2)
MAG11488 Bacteroides fragilis
MAG07010 Bacteroides fragilis_A
MAG06141 Bacteroides fragilis_A
MAG01068 Bacteroides fragilis_A
MAG07022 Bacteroides fragilis_A
MAG04711 Phocaeicola massiliensis
MAG06802 Phocaeicola massiliensis
(n=2)
MAG04572 Phocaeicola vulgatus
MAG05053 Phocaeicola vulgatus
(n=2)
MAG07417 Phocaeicola dorei
MAG05417 Bacteroides intestinalis
MAG03790 Bacteroides intestinalis_A
MAG00159 Bacteroides intestinalis_A
(n=2)
MAG06793 Bacteroides cellulosilyticus
MAG03807 Bacteroides cellulosilyticus
MAG11064 Bacteroides cellulosilyticus
(n=3)
MAG06766 Bacteroides stercorirosoris
MAG04226 Bacteroides cutis
MAG05803 Bacteroides stercoris
MAG01439 Bacteroides uniformis
MAG06346 Bacteroides uniformis
MAG06577 Bacteroides uniformis
MAG02078 Bacteroides uniformis
MAG05985 Bacteroides uniformis
MAG02633 Bacteroides uniformis
MAG06773 Bacteroides uniformis
MAG04070 Bacteroides uniformis
MAG00462 Bacteroides uniformis
MAG07332 Bacteroides uniformis
MAG10253 Bacteroides uniformis
MAG01236 Bacteroides uniformis
(n=12)
MAG04412 Parabacteroides merdae
MAG00724 Dysgonomonas capnocytophagoides
MAG07155 Bifidobacterium breve
MAG07726 Morganella morganii
MAG11544 Serratia liquefaciens
MAG11578 Serratia marcescens_I
MAG04941 Escherichia coli
MAG07959 Klebsiella_A michiganensis
MAG00708 Klebsiella_B aerogenes
MAG05052 Klebsiella quasipneumoniae
MAG03769 Klebsiella pneumoniae
MAG05218 Enterobacter roggenkampii
MAG05295 Enterobacter roggenkampii
(n=2)
MAG03097 Enterobacter himalayensis
MAG01767 Enterobacter himalayensis
(n=2)
MAG07937 Peptostreptococcus stomatis
MAG02230 Peptostreptococcus stomatis
(n=2)
MAG02201 Peptostreptococcus
MAG02310 Peptostreptococcus
MAG02357 Peptostreptococcus
MAG08065 Peptostreptococcus
(n=4)
MAG02699 GCA-900066755
MAG07423 Dorea sp000509125
MAG01687 Dorea sp000509125
MAG08992 Dorea sp000509125
(n=3)
MAG10778 Blautia hominis
MAG02492 Blautia hominis
(n=2)
MAG04219 Eisenbergiella massiliensis
MAG02487 Eisenbergiella massiliensis
MAG11490 Eisenbergiella massiliensis
MAG04401 Eisenbergiella massiliensis
(n=4)
MAG02056 Eisenbergiella sp003478085
MAG06598 Eisenbergiella tayi
MAG01511 Eisenbergiella tayi
MAG09120 Eisenbergiella tayi
(n=3)
MAG08017 Hungatella sp005845265
MAG07607 Hungatella effluvii
MAG02470 Enterocloster lavalensis
MAG01629 Enterocloster asparagiformis
MAG04328 Enterocloster citroniae
MAG00072 Enterocloster aldenensis
MAG08006 Enterocloster aldenensis
MAG05562 Enterocloster aldenensis
MAG01017 Enterocloster aldenensis
(n=4)
MAG02694 Enterocloster clostridioformis
MAG10409 Enterocloster clostridioformis
(n=2)
MAG07631 Enterocloster bolteae
MAG06898 Enterocloster bolteae
MAG05404 Enterocloster bolteae
MAG06979 Enterocloster bolteae
MAG04280 Enterocloster bolteae
MAG05059 Enterocloster bolteae
MAG04195 Enterocloster bolteae
(n=7)
MAG02161 Bulleidia
MAG11309 Faecalicoccus pleomorphus
MAG10897 Longicatena innocuum
MAG10857 Longicatena caecimuris
MAG10114 Longicatena caecimuris
MAG10775 Longicatena caecimuris
(n=3)
MAG05418 Staphylococcus aureus
MAG02175 Gemella
MAG02831 Gemella
(n=2)
MAG11559 Dolosigranulum pigrum
MAG11549 Dolosigranulum pigrum
MAG11567 Dolosigranulum pigrum
(n=3)
MAG01880 Granulicatella
MAG02212 Granulicatella sp001071995
MAG02172 Granulicatella
MAG02294 Granulicatella
(n=4)
MAG01490 Limosilactobacillus fermentum
MAG05533 Ligilactobacillus salivarius
MAG10039 Lacticaseibacillus paracasei
MAG05990 Lacticaseibacillus rhamnosus
MAG00087 Lacticaseibacillus rhamnosus
(n=2)
MAG08376 Vagococcus sp003462485
MAG02049 Enterococcus_A avium
MAG01895 Enterococcus_A avium
(n=2)
MAG00722 Enterococcus_D gallinarum
MAG00702 Enterococcus_D casseliflavus
MAG00599 Enterococcus_B faecium_B
MAG06389 Enterococcus_B faecium
MAG04596 Enterococcus faecalis
MAG10874 Enterococcus faecalis
MAG09198 Enterococcus faecalis
(n=3)
MAG02816 Lactococcus piscium_C
MAG07483 Lactococcus lactis
MAG09216 Lactococcus garvieae_B
MAG10478 Lactococcus petauri
MAG01761 Streptococcus gordonii
MAG02235 Streptococcus sanguinis
MAG11115 Streptococcus constellatus
MAG07938 Streptococcus constellatus
(n=2)
MAG05980 Streptococcus anginosus_C
MAG10855 Streptococcus anginosus
MAG09220 Streptococcus anginosus_C
MAG00798 Streptococcus anginosus_C
(n=3)
MAG03073 Streptococcus sp000479315
MAG02305 Streptococcus sp000479315
MAG08551 Streptococcus sp000479315
MAG02296 Streptococcus sp000479315
(n=4)
MAG03879 Streptococcus parasanguinis_B
MAG00710 Streptococcus parasanguinis_B
MAG05648 Streptococcus parasanguinis_B
MAG03583 Streptococcus parasanguinis_B
MAG01535 Streptococcus parasanguinis_B
MAG04278 Streptococcus parasanguinis_B
MAG03750 Streptococcus parasanguinis_B
MAG00726 Streptococcus parasanguinis
MAG10900 Streptococcus parasanguinis_B
MAG01021 Streptococcus parasanguinis_B
(n=9)
Phylum
Actinobacteriota
Bacteroidota
Firmicutes
Firmicutes_A
Proteobacteria

Fig. S21. The phylogenetic tree of strains (nrMAGs) that have the potential to use the pentose phosphate pathway (oxidative phase, glucose 6P => ribulose 5P, M00006, module completeness: 100%). The phylogenetic tree of nrMAGs was constructed using PhyloPhlAn and visualized using iTOL. The color of cycle and clades represents phylum.

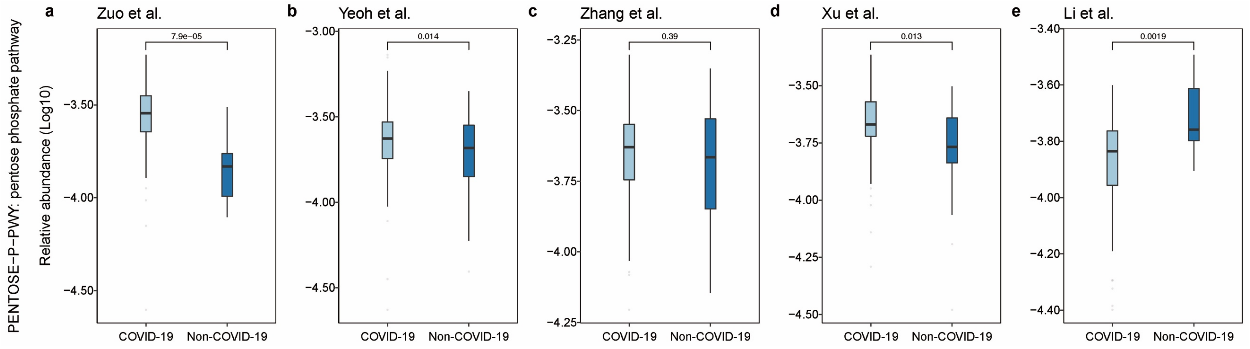

Fig. S22 Abundance comparison of the pentose phosphate pathway between COVID-19 patients and Non-COVID-19 controls. The cohort from the study of Zuo et al. (**a**, sample size n = 65), Yeoh et al. (**b**, sample size n = 274), Zhang et al. (**c**, sample size n = 198), Xu et al. (**d**, sample size n = 69), and Li et al. (**e**, sample size n = 65). *P* values were calculated by two-sided Wilcoxon–Mann–Whitney test. Boxplots with medians are shown; the lower and upper hinges correspond to the first and third quartiles (the 25th and 75th percentiles); the upper and lower whiskers extend from the hinge to the largest and smallest value no further than 1.5× interquartile range from the hinge; outliers are plotted by translucent circles.

Table S1: Validation cohorts analyzed in this study. COVID-19 negative samples are healthy controls or Non-COVID-19 patients who tested negative for SARS-CoV-2 infection.

**Dataset**	**Zhang et al.**	**Xu et al.**	**Li et al.**	**Total**
COVID-19 positive samples	129	38	46	213
COVID-19 negative samples	78	31	19	128
Total subjects	144	69	65	278
Total samples	207	69	65	341
Longitudinal	Yes	No	No	-
Source	Feces	Feces	Feces	-
Geography	CHINA	CHINA	CHINA	-
Year	2022	2022	2021	
Sequencing platform	Illumina NovaSeq 6000	Illumina NovaSeq 6000	BGISEQ-500 platform	-
Total sequences (mean)	23,449,564.35	34,340,178.93	38,464,386.58	-
Accession number	PRJNA689961	SRP118759 & PRJNA792726	PRJEB43555	-
