# Supplementary material for: Dissecting the role of the human microbiome in COVID-19 via metagenome-assembled genomes
Source: Nat Commun. 2022 Sep 6;13:5235. doi: 10.1038/s41467-022-32991-w (PMC9446638; doi:10.1038/s41467-022-32991-w)
Supplement: Supplementary file 3 — Description of Additional Supplementary Files [file 41467_2022_32991_MOESM3_ESM.docx]

**Description of Additional Supplementary Files**

File Name: Supplementary Data 1

Description: COVID-19 disease severity is associated with the strain richness of certain species. (Uploaded as a separate excel file).

File Name: Supplementary Data 2

Description: Putatively permissive and protective nrMAGs of SARS-CoV-2 infection identified from the study of Yeoh et al. (Uploaded as a separate excel file).
