# Supplementary material for: Dissecting the role of the human microbiome in COVID-19 via metagenome-assembled genomes
Source: Nat Commun. 2022 Sep 6;13:5235. doi: 10.1038/s41467-022-32991-w (PMC9446638; doi:10.1038/s41467-022-32991-w)
Supplement: Supplementary file 6 — Reporting Summary [file 41467_2022_32991_MOESM6_ESM.pdf]

nature portfolio

Corresponding author(s): Yang-Yu Liu

Last updated by author(s): Aug 21, 2022

Reporting Summary

Nature Portfolio wishes to improve the reproducibility of the work that we publish. This form provides structure for consistency and transparency in reporting. For further information on Nature Portfolio policies, see our Editorial Policies and the Editorial Policy Checklist.

Statistics

For all statistical analyses, confirm that the following items are present in the figure legend, table legend, main text, or Methods section.

n/a	Confirmed	
☐	☒	The exact sample size (*n*) for each experimental group/condition, given as a discrete number and unit of measurement
☐	☒	A statement on whether measurements were taken from distinct samples or whether the same sample was measured repeatedly
☐	☒	The statistical test(s) used AND whether they are one- or two-sided<br>*Only common tests should be described solely by name; describe more complex techniques in the Methods section.*
☐	☒	A description of all covariates tested
☐	☒	A description of any assumptions or corrections, such as tests of normality and adjustment for multiple comparisons
☐	☒	A full description of the statistical parameters including central tendency (e.g. means) or other basic estimates (e.g. regression coefficient) AND variation (e.g. standard deviation) or associated estimates of uncertainty (e.g. confidence intervals)
☐	☒	For null hypothesis testing, the test statistic (e.g. *F*, *t*, *r*) with confidence intervals, effect sizes, degrees of freedom and *P* value noted<br>*Give P values as exact values whenever suitable.*
☒	☐	For Bayesian analysis, information on the choice of priors and Markov chain Monte Carlo settings
☒	☐	For hierarchical and complex designs, identification of the appropriate level for tests and full reporting of outcomes
☐	☒	Estimates of effect sizes (e.g. Cohen's *d*, Pearson's *r*), indicating how they were calculated

Our web collection on statistics for biologists contains articles on many of the points above.

Software and code

Policy information about availability of computer code

Data collection	The sequencing data were downloaded from online repositories (i.e., NCBI, ENA, and GSA). No software was used for the data collection.
Data analysis	Genome reconstruction of human microbiome with metagenomic sequencing data was performed with the function modules of metaWRAP (v1.3.2), including<br>metaWRAP-Read_qc module (quality control),<br>metaWRAP-Assembly module (assembly) using metaSPAdes (v3.13.0),<br>metaWRAP-Binning (binning) with MaxBin2 (v2.2.6), metaBAT2 (v2.12.1), and CONCOCT (v1.0.0),<br>metawraps-Bin_refinement (Refinement of MAGs)<br>CheckM (v1.0.12) was used to estimate the quality of the bins.<br><br>The genome annotations of MAGs were performed with Prokka (v1.13) and MicrobeAnnotator (v2.0.5). Functional profiling was performed using HUMANN3(v3.0.1) together with Bowtie2 (v2.4.5), UniRef90 (v201901b), and DIAMOND (v2.0.15).<br><br>Details information are provided in the methods section of the manuscript. The project related specific codes are available at: https://github.com/Owenke247/COVID-19 or https://doi.org/10.5281/zenodo.6824864.

For manuscripts utilizing custom algorithms or software that are central to the research but not yet described in published literature, software must be made available to editors and reviewers. We strongly encourage code deposition in a community repository (e.g. GitHub). See the Nature Portfolio guidelines for submitting code & software for further information.

Data

Policy information about availability of data

All manuscripts must include a data availability statement. This statement should provide the following information, where applicable:

- Accession codes, unique identifiers, or web links for publicly available datasets
- A description of any restrictions on data availability
- For clinical datasets or third party data, please ensure that the statement adheres to our policy

All data used in this article come from publicly available sources. The metagenomic data from the discovery cohorts are available in the NCBI or Genome Sequence Archive Bioproject database under accession code PRJNA624223 (https://www.ncbi.nlm.nih.gov/bioproject/?term=PRJNA624223), PRJNA656660 (https://www.ncbi.nlm.nih.gov/bioproject/?term=PRJNA656660), PRJNA660883 (https://www.ncbi.nlm.nih.gov/bioproject/?term=PRJNA660883), PRJNA743981 (https://www.ncbi.nlm.nih.gov/bioproject/?term=PRJNA743981), PRJCA003532 (https://ngdc.cncb.ac.cn/gsa/browse/CRA003271), and PRJNA650244 (https://www.ncbi.nlm.nih.gov/bioproject/?term=PRJNA650244). The metagenomic data from the validation cohorts are available in the NCBI Bioproject or SRA database under accession code PRJNA689961 (https://www.ncbi.nlm.nih.gov/bioproject/?term=PRJNA689961), SRP118759 (https://www.ncbi.nlm.nih.gov/sra/?term=SRP118759), PRJNA792726 (https://www.ncbi.nlm.nih.gov/bioproject/?term= PRJNA792726), PRJEB43555 (https://www.ncbi.nlm.nih.gov/bioproject/?term=PRJEB43555). Metagenome-assembled genomes for all samples are available on Figshare (https://figshare.com/s/a426a12b463758ed6a54). HUMANN3 databases for metagenomic functional profiling were accessed from http://huttenhower.sph.harvard.edu/humann_data/.

Field-specific reporting

Please select the one below that is the best fit for your research. If you are not sure, read the appropriate sections before making your selection.

☒ Life sciences ☐ Behavioural & social sciences ☐ Ecological, evolutionary & environmental sciences

For a reference copy of the document with all sections, see nature.com/documents/nr-reporting-summary-flat.pdf

Life sciences study design

All studies must disclose on these points even when the disclosure is negative.

Sample size	We identified COVID-19 metagenomic sequencing studies from keyword searches in PubMed and online repositories (i.e., NCBI, ENA, and GSA) and by following references in meta-analyses and related microbiome studies. We included samples with publicly available raw shotgun metagenomic sequencing data (paired fastq files) and metadata indicating patients with COVID-19 or Non-COVID-19 control status. All the sequencing data were downloaded from online repositories or links provided in the original publications, but some metadata were acquired after personal communication with the authors. We did not include any studies which required additional ethics committee approvals or authorizations for access. A total of 514 and 341 microbiome samples from two discovery cohorts and three validation cohorts were analyzed in this study, respectively.
Data exclusions	We excluded samples without publicly available raw shotgun metagenomic sequencing data (paired fastq files) and metadata indicating patients with COVID-19 or Non-COVID-19 control status.
Replication	Reproducibility of data and results is ensured by performing internal program and technical review. Reproducibility of published datasets can be found from the following original papers:<br>Zuo et al., Alterations in Gut Microbiota of Patients With COVID-19 During Time of Hospitalization.<br>Britton et al., Limited intestinal inflammation despite diarrhea, fecal viral RNA and SARS-CoV-2-specific IgA in patients with acute COVID-19.<br>Cao et al., Integrated gut virome and bacteriome dynamics in COVID-19 patients.<br>Yeoh et al., Gut microbiota composition reflects disease severity and dysfunctional immune responses in patients with COVID-19.<br>Liu et al., Association between the nasopharyngeal microbiome and metabolome in patients with COVID-19.<br>Zhang et al., Prolonged Impairment of Short-Chain Fatty Acid and L-Isoleucine Biosynthesis in Gut Microbiome in Patients With COVID-19.<br>Xu et al., Integrated analysis of gut microbiome and host immune responses in COVID-19<br>Li et al., Microbiome Profiling Using Shotgun Metagenomic Sequencing Identified Unique Microorganisms in COVID-19 Patients With Altered Gut Microbiota.<br><br>We ensured the reproducibility of our findings by performing external validations. All attempts at replication were successful.
Randomization	Not applicable. This study is not a clinical trial. There are not experimental groups and interventions in this study.
Blinding	Not applicable. This is an observational study without any interventions.

Reporting for specific materials, systems and methods

We require information from authors about some types of materials, experimental systems and methods used in many studies. Here, indicate whether each material, system or method listed is relevant to your study. If you are not sure if a list item applies to your research, read the appropriate section before selecting a response.

Materials & experimental systems

n/a	Involved in the study
☒	☐ Antibodies
☒	☐ Eukaryotic cell lines
☒	☐ Palaeontology and archaeology
☒	☐ Animals and other organisms
☐	☒ Human research participants
☒	☐ Clinical data
☒	☐ Dual use research of concern

Methods

n/a	Involved in the study
☒	☐ ChIP-seq
☒	☐ Flow cytometry
☒	☐ MRI-based neuroimaging

Human research participants

Policy information about studies involving human research participants

Population characteristics	This study population included 657 participants from 9 public available metagenomic datasets. The detailed population characteristics can be found in the original paper.
Recruitment	The recruitment information can be found in the original paper.
Ethics oversight	We did not include any studies which required additional ethics committee approvals or authorizations for access. The ethics approval can be found in the original paper.

Note that full information on the approval of the study protocol must also be provided in the manuscript.
